# Global prevalence and phylogeny of hepatitis B virus (HBV) drug and vaccine resistance mutations

**DOI:** 10.1111/jvh.13525

**Published:** 2021-05-07

**Authors:** Jolynne Mokaya, Tetyana I. Vasylyeva, Eleanor Barnes, M. Azim Ansari, Oliver G. Pybus, Philippa C. Matthews

**Affiliations:** ^1^ Nuffield Department of Medicine Oxford UK; ^2^ Division of Infectious Diseases & Global Public Health Department of Medicine University of California San Diego CA USA; ^3^ Department of Hepatology Oxford University Hospitals NHS Foundation Trust John Radcliffe Hospital Oxford UK; ^4^ National Institutes of Health Research Health Informatics Collaborative NIHR Oxford Biomedical Research Centre John Radcliffe Hospital Oxford UK; ^5^ Wellcome Centre for Human Genetics Oxford UK; ^6^ Department of Zoology University of Oxford Oxford UK; ^7^ Department of Infectious Diseases and Microbiology Oxford University Hospitals NHS Foundation Trust John Radcliffe Hospital Oxford UK

**Keywords:** Africa, entecavir, epidemiology, HBV, HBV vaccine, lamivudine, NAs, NUCs, prevention, RAMs, resistance, TDF, tenofovir, therapy

## Abstract

Vaccination and anti‐viral therapy with nucleos(t)ide analogues (NAs) are key approaches to reducing the morbidity, mortality and transmission of hepatitis B virus (HBV) infection. However, the efficacy of these interventions may be reduced by the emergence of drug resistance‐associated mutations (RAMs) and/or vaccine escape mutations (VEMs). We have assimilated data on the global prevalence and distribution of HBV RAMs/VEMs from publicly available data and explored the evolution of these mutations. We analysed sequences downloaded from the HBV Database and calculated prevalence of 41 RAMs and 38 VEMs catalogued from published studies. We generated maximum likelihood phylogenetic trees and used treeBreaker to investigate the distribution and estimated the age of selected mutations across tree branches. RAM M204I/V had the highest prevalence, occurring in 3.8% (109/2838) of all HBV sequences in our data set, and a significantly higher rate in genotype C at 5.4% (60/1102, *p* = 0.0007). VEMs had an overall prevalence of 1.3% (37/2837) and had the highest prevalence in genotype C and in Asia at 2.2% (24/1102; *p *= 0.002) and 1.6% (34/2109; *p *= 0.009), respectively. Phylogenetic analysis suggested that RAM/VEMs can arise independently of treatment/vaccine exposure. In conclusion, HBV RAMs/VEMs have been found globally and across genotypes, with the highest prevalence observed in genotype C. Screening for genotype and for resistance‐associated mutations may help to improve stratified patient treatment. As NAs and HBV vaccines are increasingly being deployed for HBV prevention and treatment, monitoring for resistance and advocating for better treatment regimens for HBV remains essential.

**Abbreviations**
BEAST: Bayesian Evolutionary Analysis Sampling Trees
ETV: entecavir
HBV: hepatitis B virus
HCC: hepatocellular cancer
HCV: hepatitis C virus
ML: maximum likelihood
NAs: nucleos(t)ide analogues
RAMs: resistance‐associated mutations
RT: reverse transcriptase
TDF: tenofovir disoproxil fumarate
TFV: Tenofovir
TMRCA: time to most recent common ancestor
VEMs: vaccine escape mutations

**Significance statement**

Vaccination and anti‐viral therapy with nucleos(t)ide analogues (NAs) are key approaches to reducing the burden of hepatitis B virus (HBV) infection. However, the efficacy of these interventions may be reduced by the emergence of mutations associated with drug and vaccine resistance. We have assimilated data on the global prevalence and distribution of these mutations. We report that resistance‐associated mutations are present globally and across genotypes, with the highest prevalence observed in genotype C. As NAs and HBV vaccines are increasingly being deployed for HBV prevention and treatment, monitoring for resistance and advocating for better treatment regimens for HBV remains essential.

## 1 INTRODUCTION

Anti‐viral therapy with nucleos(t)ide analogue (NA) agents is a central approach to reducing morbidity, mortality and transmission of hepatitis B virus (HBV) infection. NAs are used to suppress viral replication, thus reducing inflammatory liver damage.^1^ However, the efficacy of widespread deployment of NAs, both for individual patients and at a public health level, may be affected by the emergence of drug resistance.^2^, ^3^ Resistance‐associated mutations (RAMs) can arise as a result of the low fidelity of the HBV reverse transcriptase (RT) enzyme which lacks transcriptional proofreading activity, especially relevant in the setting of high viral replication rates (estimated at up to ~10^12^ virions/day^2^).

Lamivudine (3TC) and entecavir (ETV) were licensed in 1986 and 2005, respectively, but their ongoing role has been variably limited by anti‐viral drug resistance.^4^, ^5^, ^6^ Tenofovir (TFV), most commonly prescribed as tenofovir disoproxil fumarate (TDF), was licensed in 2008 and is now the favoured choice as it has a higher genetic barrier to resistance, as well as being cheap, well‐tolerated and safe, including in pregnancy.^4^ However, there are now emerging data that show the potential for selection of TDF drug resistance mutations,^7^ albeit with limited insights into the prevalence or clinical impact of these RAMs.^8^ Importantly, as well as being selected in individuals on therapy, RAMs have been reported among treatment‐naïve individuals.^1^, ^9^ Whether these mutations occur without exposure to anti‐virals, or are exclusively as result of prior drug exposure, is uncertain.

Reports of resistance to the HBV vaccine raise concerns about the extent to which vaccine‐mediated immunity will remain robust. The vaccine, licensed for use in 1981, is administered to infants as part of WHO expanded programme for immunization.^10^ HBV vaccination induces the production of neutralizing antibodies that mainly target the second hydrophilic loop (amino acids (aa) 139 to 147 or 149) of the major antigenic determinant (aa 99 to 169) of the HBV surface protein (HBsAg).^11^, ^12^ Immune pressure can lead to the selection of mutations within HBsAg, resulting in variants resistant to HBV vaccine and/or HBV immunoglobulin (HBIg).^2^ G145A/R is the best described mutation associated with resistance to HBV vaccine/HBIg.^11^, ^12^, ^13^ Several other mutations across the entire antigenic determinant have been reported, which also have associations with vaccine resistance.^11^, ^14^, ^15^, ^16^

Genetic differences among the ten HBV genotypes (A–J) and numerous sub‐genotypes may influence the likelihood of acquisition of drug or vaccine resistance.^17^ Genotypes have different geographical distributions, for example genotypes A, D and E are predominant in Africa, and B and C in Asia.^18^, ^19^ In genotypes in which the wild‐type amino acid at a specific position is part of a sequence motif associated with drug or vaccine resistance, the barrier to resistance is likely to be inherently lower. This phenomenon has been described in hepatitis C virus (HCV) infection, explaining why some sub‐genotypes are intrinsically resistant to the most widely used direct‐acting anti‐viral drugs.^20^, ^21^, ^22^ In addition, genotype‐specific differences in mutation rates and host population dynamics have an influence on virus evolutionary rates, which directly affects the probability of appearance of RAMs/VEMs. For HBV, the rate of molecular evolution is estimated to be between 7.9 × 10^−5^ and 3.2 × 10^−4^ substitutions per site per year.^23^, ^24^

A number of studies have reported the frequencies of RAMs in HBV from different populations^1^, ^25^, ^26^, ^27^; however, the global prevalence, geographic distribution, time of origins and their association with different HBV genotypes remain unknown. We therefore set out to assimilate data on the global prevalence and distribution of HBV RAMs from public sequence databases, and to explore the genetic relatedness of viruses bearing these mutations.

## 2 METHODS

### 2.1 HBV sequence curation process

We analysed sequences downloaded from a publicly available database (Hepatitis B Virus Database—https://hbvdb.ibcp.fr/HBVdb/ ^28^), accessed on 20th November 2018. We downloaded a total of 6219 full‐length genome sequences (Figure S1). Using MEGA7 software,^29^ we generated neighbour‐joining phylogenetic trees to validate the HBV genotype assignment, discarding sequences that had been incorrectly classified. We then generated pairwise distances for aligned sequences within each genotype using the dist.alignment function of the R package seqinr,^30^ and excluded sequences with >99.5% similarity in order to remove closely related isolates (for instance, duplicates and/or isolates derived from the same individual). For the remaining sequences, we obtained sample collection date and sampling country from GenBank. A total of 2938 sequences had geographical data and 2167 had both sample collection date and geographical data. Figure S1 shows the data curation process.

### 2.2 Drug resistance‐associated mutations

We worked from a list of pre‐existing drug RAMs identified from published studies^1^, ^2^, ^8^, ^9^, ^25^, ^31^ (Table S1), and stratified them according to the NA to which they cause resistance, as described below:

1. 3TC resistance: We classified RAMs associated with 3TC into three categories: (i) primary RAMs, which are well known to cause resistance to 3TC in isolation; (ii) compensatory RAMs, which by themselves do not confer resistance but when combined with primary RAMs enhance resistance and viral functional capacity^2^; and (iii) putative RAMs for which there is limited clinical/phenotypic evidence for 3TC resistance.
2. ETV resistance: Two or more amino acid substitutions are required across the HBV RT protein to confer resistance to ETV which could occur as a combination of M204I/V with one or more of the following substitutions L80I/V, I163V, I169T, V173L, L180M, A181S/T/V, T184X, A186T, S202C/G/I/R, M250I/V and/or C256S/G.
3. TFV resistance: We classified RAMs associated with TFV into three categories: those with both clinical and in vitro evidence; those with only phenotypic evidence; and those with only experimental evidence, as described in a systematic literature review.^8^

### 2.3 Vaccine escape mutations

All pre‐existing VEMs included in this study were identified from published studies^1^, ^14^, ^15^, ^16^, ^32^, ^33^, ^34^, ^35^, ^36^, ^37^, ^38^, ^39^(Table S2). K141E/I/R and G145A/R have the strongest evidence base of clinical and in vitro data to support HBV vaccine resistance,^33^ while other VEMs are considered putative, as they are supported by less robust data.

### 2.4 Prevalence analyses

For the global prevalence analysis, we included HBV sequences with known country of origin from genotypes A–E; we excluded genotypes F, G & H from this analysis because of low sample size (<100), resulting in a total of 2838 sequences. For all polymorphisms that have been reported in association with resistance listed in Table S1 and S2, we calculated the prevalence (=total number of sequences with a specified mutation/total number of sequences for that genotype and continent).

We carried out prevalence analysis reporting confidence intervals and *p*‐values for individual RAMs common to 3TC, ETV and TFV, for individual or combined RAMs associated with ETV and TFV resistance, and for individual VEMs. We calculated confidence intervals using an online software Epitools (http://epitools.ausvet.com.au). We used a chi‐squared test to compare the prevalence of RAMs/VEMs between different genotypes and between different continents. We used GraphPad Prism v7.0 for data visualization and statistical analyses.

### 2.5 Distribution of selected RAMs and VEMs on maximum likelihood phylogenetic trees

We generated maximum likelihood (ML) phylogenetic trees for HBV genotype A (*n* = 290), B (*n* = 730), C (*n* = 1102), D (*n* = 565) and E (*n* = 150) sequences for which geographic information was available. We used the general time‐reversible nucleotide substitution model with gamma‐distributed among‐site rate variation (GTR + G) in IQ‐TREE.^40^ We chose this model as it incorporates different rates for every nucleotide change and different nucleotide frequencies, thus allowing for most flexibility allowing us to avoid a model‐selection step.^41^ We rooted the trees using the mid.point function of the R package phangorn.^42^

For this analysis, we considered a total of 12 RAMs (S106C/G, D134E, R153W/Q, V173L, L180M, A181T/V, A194T, A200V, M204I/V, L217R, L229V/W and I269L). These RAMs were selected because they are primary RAMs or have robust evidence in causing resistance to 3TC, ETV and/or TFV. We also considered eight VEMs (C139S, S/T140I, P142S, S/T143L/M, D144A/E/G/N, G145A/E/R, K141A/I/R and C147S) which are located within the epitope region neutralized by the HBV vaccine (aa139–147).

We used treeBreaker^43^ to determine whether sequences with individual mutations were randomly distributed across the branches of the phylogenetic trees reconstructed for each genotype. The program calculates per‐branch posterior probability of having a change in the distribution of a discrete character and gives a Bayes factor to show the strength of this evidence.^43^ A Bayes factor of >30 indicates strong evidence that sequences with RAMs/VEMs are not randomly distributed on a phylogenetic tree. We performed this analysis for each mutation separately.

### 2.6 Phylogenetic dating

We performed phylogenetic dating to estimate the times of emergence of mutations of interest, focused on RAMs V173L, L180M and M204I/V as they are best recognized to cause or contribute (individually or synergistically) resistance to 3TC, ETV and TDF,^8^ and VEMs G145A/R and K141E/I/R as they have been best described to cause HBV vaccine resistance.^11^, ^12^, ^13^ In this analysis, we included genotypes that had >50 sequences with associated sampling date information: genotype A (*n* = 170), B (*n* = 594), C (*n* = 906), D (*n* = 336) and E (*n* = 88). We manually inspected sequences for misalignments using AliView^43^ and then excluded codon positions associated with resistance (we excluded all sites listed in Tables S1 and S2) to ensure that parallel evolution of RAMs/VEMs does not affect the phylogeny.^44^ We used ML phylogenetic trees generated for each genotype as described above and then dated these phylogenetic trees using IQ‐TREE v2.0.3.^45^ We resampled the trees 100 times and chose the lognormal relaxed molecular clock model because it has performed best in other studies of HBV evolution.^46^, ^47^ We used TempEst to estimate the molecular clock signal in our data sets by regressing the root‐to‐tip genetic distance of each sequence in the tree and its sampling date.^48^ Based on application of TempEst, we estimated the correlation between the dates of the tips of the sequences and the divergence from the root to be 7.8 × 10^−2^, 3.9 × 10^−1^, 4.3 × 10^−2^, 2.3 × 10^−2^ and 2.1 × 10^−1^ for genotypes A, B, C, D and E, respectively. Due to the lack of correlation, we used the substitution rate estimated before^24^, ^49^ and therefore we fixed the mean substitution rate to 5.0 × 10^−5^ (SD 4.12 × 10^−6^) subs/site/year for all genotypes in all subsequent analyses. We reported the time to most recent common ancestor (TMRCA) of two or more sequences that clustered together having the same mutation as this TMRCA likely corresponds to the lower bound of the age of the mutation.

We also performed molecular clock phylogenetic analyses using Bayesian Evolutionary Analysis Sampling Trees (BEAST). This method is described in Supplementary Methods.

## 3 RESULTS

### 3.1 Global prevalence of HBV drug RAMs

We assessed the prevalence of polymorphisms associated with drug resistance across 48 different sites within RT protein in a total of 2838 full‐length HBV sequences, Table S1. 90% (43/48) of the sites of interest had polymorphisms associated with drug resistance, Figure S2. 60% of the sites had polymorphisms associated with drug resistance occurring at a prevalence of between 0% and 10% in both genotypes and continents. Genotypes A and C, as well as sequences from Europe, had the highest number of sites (nine sites for genotype A and C, and 11 sites for Europe) with polymorphisms associated with drug resistance occurring at a prevalence of >20%, Figure S2.

### 3.2 RAMs common to 3TC, ETV and/or TDF

**FIGURE 1:**
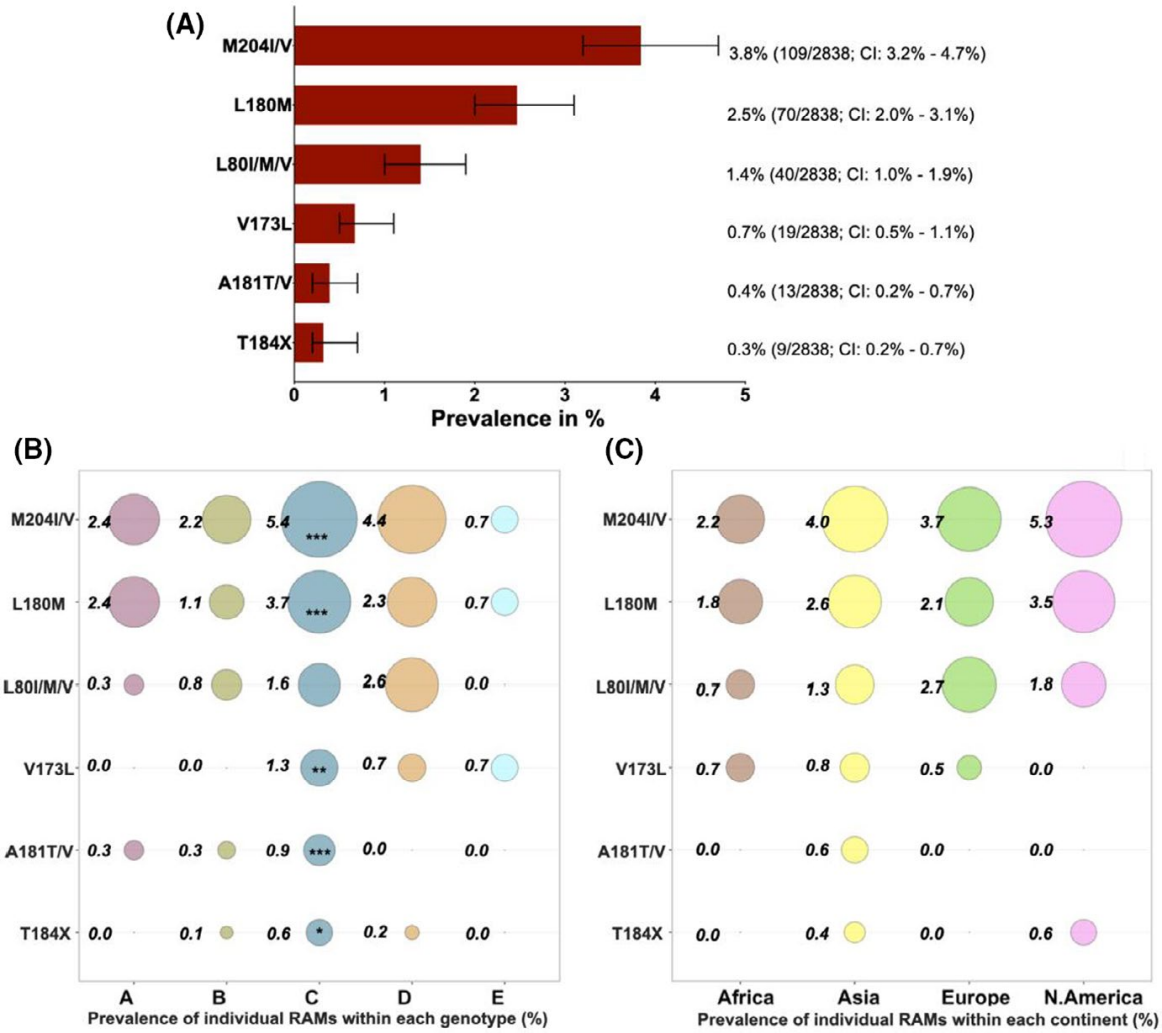
Global prevalence of hepatitis B virus (HBV) drug resistance‐associated mutations (RAMs) obtained from analysing 2838 HBV sequences with information on country of origin, downloaded from a public database (https://hbvdb.ibcp.fr/HBVdb/). (A) Overall prevalence of RAMs common to 3TC, ETV and TFV. (B) A bubble plot showing the overall prevalence of RAMs common to 3TC, ETV and TFV within each genotype (genotype A *n* = 290; Genotype B *n* = 730; Genotype C *n* = 1102; Genotype D *n* = 566; Genotype E *n* = 150). (C) A bubble plot showing the overall prevalence of RAMs common to 3TC, ETV and TFV within each continent (Africa *n* = 277; Asia *n* = 2109; Europe *n* = 187; North America *n* = 170). Numbers next to the circles are prevalence (%) of individual RAMs in each genotype/continent. The asterisks (***/**/*) within certain circles indicate RAMs that have a higher prevalence within the specified genotype/continent compared to the prevalence of that RAM in other genotypes/continents and is statistically significant. ***p value < 0.001; **p value < 0.005; *p value < 0.05. Bars show 95% confidence intervals. T184X represents T184A/C/F/G/I/L/M/S

RAMs L80I/M/V, V173L, L180M, A181T/V and T184X are common to 3TC, ETV and/or TFV. M204I/V had the highest overall prevalence at 3.8% (109/2838) (Figure 1A). Genotype C had the highest prevalence of all of these mutations, apart from L80I/M/V which is most common in Genotype D (although the difference is not statistically significant) (Figure 1B). L180M and M204I/V were both present in all genotypes and continents that we analysed (Figure 1B, C). However, there were no significant differences in prevalence of these RAMs across continents.

### 3.3 RAMs associated with ETV resistance

The overall prevalence of ETV resistance predicted by this data set, determined by the presence of RAMs M204I/V+L180M, was 2.4% (67/2838); other combinations of ETV drug‐resistant mutations were uncommon (all <0.6%); Figure S3. As previously, the most common resistance mutations were seen in genotype C at 3.5% (39/1102 vs. 28/1736 in other genotypes; *p *= 0.001).

### 3.4 RAMs associated with TFV resistance

The prevalence of individual mutations that have been associated with TFV resistance ranged between 0.2 and 19.5%. Compared to all other genotypes, genotype C had the highest prevalence of individual RAMs S106C/G, DH/N134E and I269L, and Asia had the highest prevalence of these individual RAMs S106C/G, DH/N134E and I269L compared to other continents, Figure S4.

However, isolated RAMs are unlikely to cause TFV resistance, and combinations of RAMs are likely to be required to confer clinically significant TFV resistance.^8^ We therefore sought evidence of these combinations of mutations in our sequence database (*n* = 2838). In each case, we only identified between one and three sequences with each combination of RAMs giving an overall prevalence of between 0.04% and 0.1% (Table S3), suggesting these arise infrequently and are currently unlikely to be of significance at a population level. The majority of sequences carrying these drug resistance motifs were again in genotype C.

### 3.5 Global prevalence of VEMs

We investigated the prevalence of polymorphisms associated with vaccine/HBIg escape across 33 different sites within surface protein in a total of 2838 full‐length HBV sequences, Table S2. 78% (25/33) sites had polymorphisms associated with vaccine/HBIg escape, Figure S5 52% (12/23) of the sites had polymorphisms associated with vaccine escape occurring at a prevalence of between 0% and 9% in both genotypes and continents. Genotype C and Asia had the highest number of sites with polymorphisms associated with vaccine escape occurring at a prevalence of >20%, compared to other genotypes and continents, Figure S5.

**FIGURE 2:**
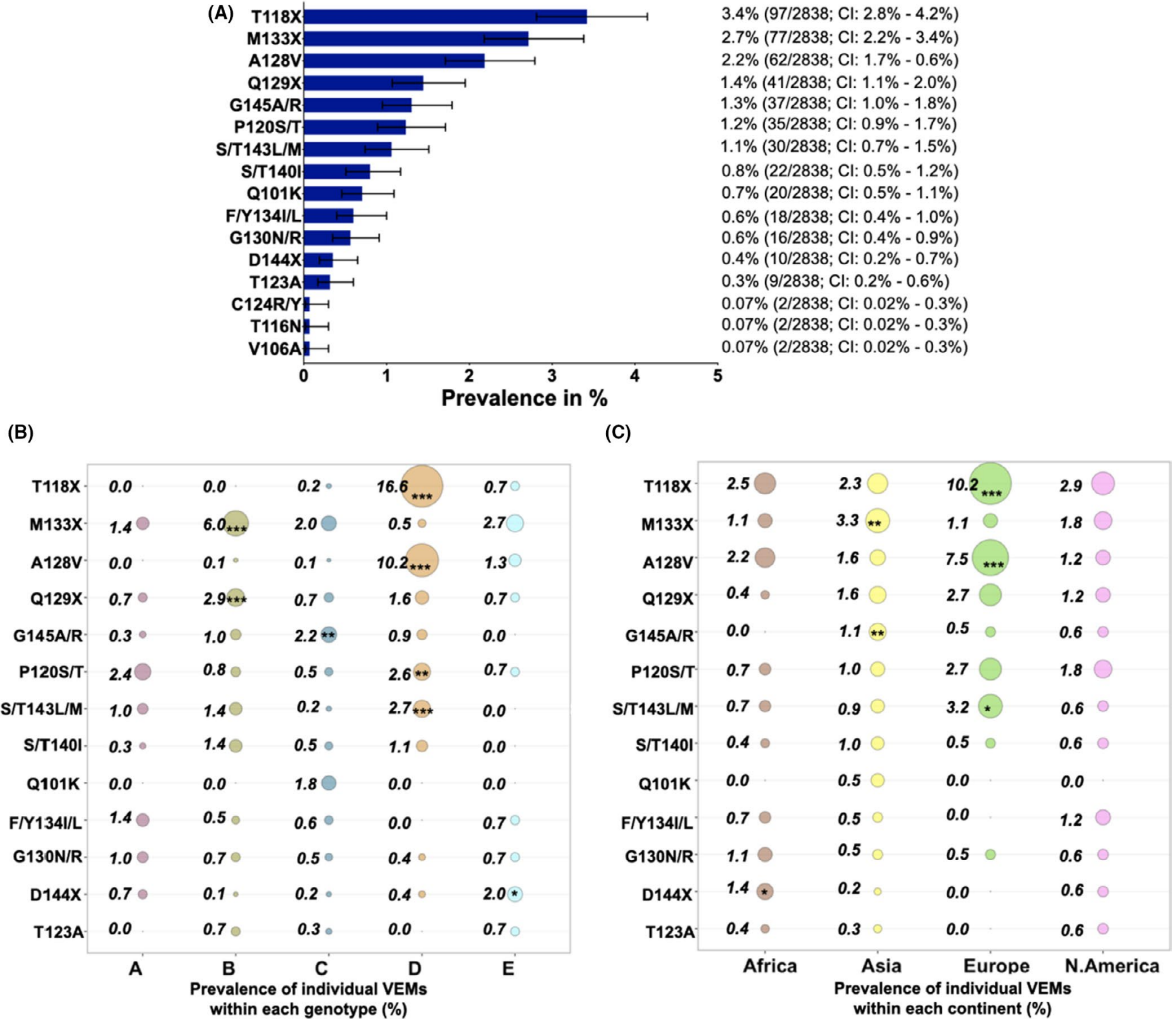
Global prevalence of hepatitis B virus (HBV) vaccine escape mutations (VEMs) obtained from analysing 2838 HBV sequences with information on country of origin, downloaded from a public database (https://hbvdb.ibcp.fr/HBVdb/). (A) Overall prevalence of putative VEMs and/or VEMs with only clinical or in vitro evidence. (B) A bubble plot showing the overall prevalence of putative VEMs and/or VEMs with only clinical or in vitro evidence within each genotype (genotype A *n* = 290; Genotype B *n* = 730; Genotype C; *n* = 1102; Genotype D *n* = 566 and Genotype E *n* = 150), with a prevalence of >0.1%. (C) A bubble plot showing the overall prevalence of putative VEMs and/or VEMs with only clinical or in vitro evidence within each continent (Africa *n* = 277; Asia *n* = 2109; Europe; *n* = 187 and North America *n* = 170), with a prevalence of >0.1%. Numbers next to the circles are prevalence (%) of individual RAMs in each genotype/continent. The asterisks (***/**/*) within certain circles indicate RAMs that have a higher prevalence within the specified genotype/continent compared to the prevalence of that RAM in other genotypes/continents and is statistically significant. ***p value < 0.001; **p value < 0.005; *p value < 0.05. T118X represents T118A/R/V; M133X represents M133I/L/T; Q129X represents Q129A/R; D144X represents D144A/E/G/N

Vaccine escape mutations K141E/I/R was not present in our data set. G145A/R had an overall prevalence of 1.3% (37/2837) and had the highest prevalence in genotype C at 2.2% (24/1102; *p *= 0.002), and in Asia at 1.6% (34/2109; *p *= 0.009); this is the best recognized VEM (Figure 2A). Other VEMs that had an overall prevalence of >1% were T118A/R/V, M133I/L/T, A128V, Q129H/N/R, G145A/R, P120S/T and S/T143L/M (Figure 2A). T118A/R/V, A128V and S/T143L/M had the highest prevalence in genotype D and in Europe, being present >3% of the sequences. M133I/L/T and Q129H/N/R had the highest prevalence in genotype B and M133I/L/T had the highest prevalence in Asia, also being present in >3% of the sequences (Figure 2B and 2C).

### 3.6 RAMs/VEMs as wild‐type amino acid

Describing the evolution, epidemiology or clinical significance of individual RAMs/VEMs in HBV sequences is difficult because some of the mutations that have been described occur at consensus level in some genotypes. For example, 11 polymorphisms associated with drug resistance and nine polymorphisms associated with vaccine escape had a prevalence of >50% in ≥1 genotype (s), Tables S4 and S5. RAM H/Y9H is wild type in genotypes A‐E. This mutation is most likely to contribute to resistance as a ‘flexible’ position in the protein, in which compensatory change is easily incorporated. RAMs H126Y and R/W153W, which contribute to TFV resistance when combined with ≥3 other RAMs,^8^ are wild type in genotype A. This observation illustrates that resistance to different drugs or HBV vaccine might be more easily selected in certain populations or regions, based on the global distribution of HBV genotypes.

### 3.7 Distribution of selected RAMs and VEMs on maximum likelihood phylogenetic trees

We considered the distribution of 12 RAMs (S106C/G, D134E, R153W/Q, V173L, L180M, A181T/V, A194T, A200V, M204I/V, L217R, L229V/W and I269L) and eight VEMs (C139S, S/T140I, P142S, S/T143L/M, D144A/E/G/N, G145A/E/R, K141A/I/R and C147S) across the branches of ML phylogenetic trees. Most of these RAMs and all VEMs were randomly distributed across the branches of phylogenetic trees reconstructed from genotypes A‐E sequences, suggesting parallel evolution.

**FIGURE 3:**
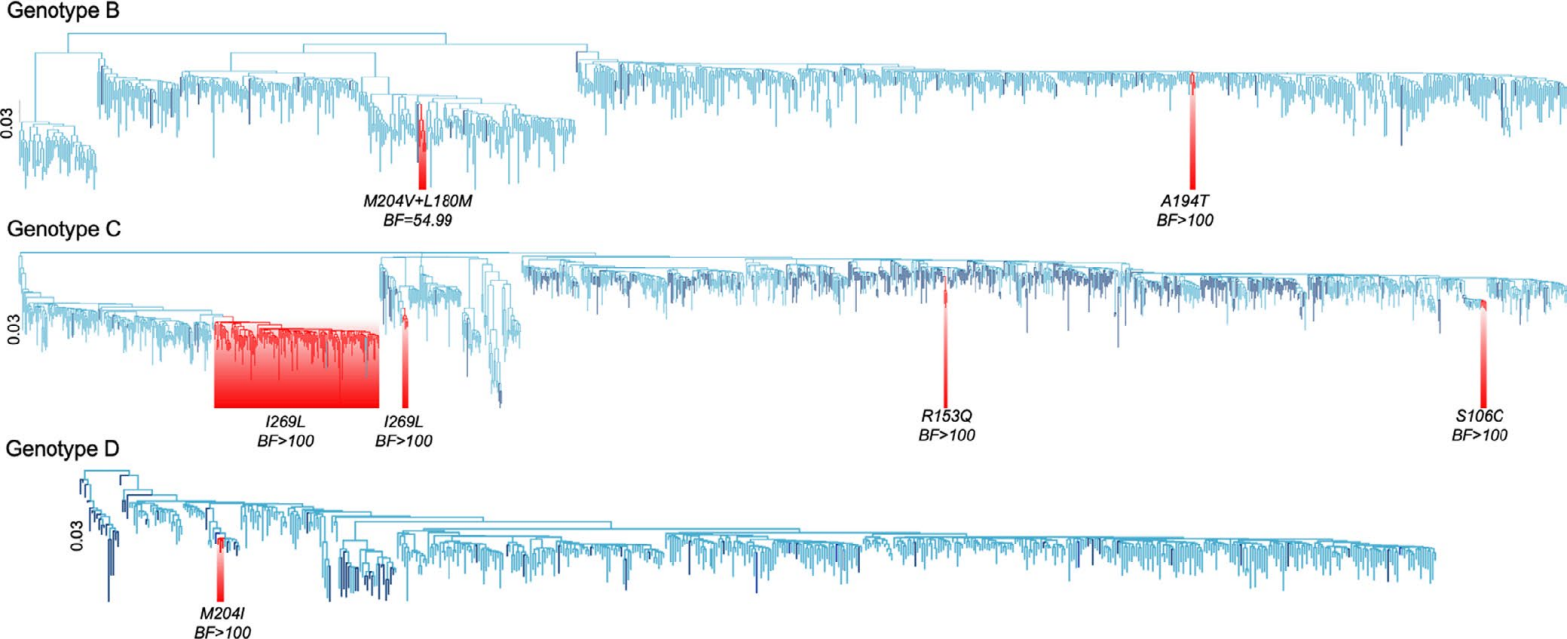
HBV RAMs/VEMs distribution on rooted maximum likelihood phylogenetic trees for genotype B, C and D. Branches in dark blue represent sequences with one or more RAMs/VEMs. Branches in light blue have no specified RAMs/VEMs. Branches highlighted in red indicate clustered sequences with a RAM with Bayes factor of >30, suggesting strong evidence of clustering. ML trees for genotype A and E were not displayed because they had no sequences with specified RAM/VEM which formed clusters

However, there were several RAMs that clustered within genotype B, C and D sequences (Figure 3). In genotype B, all sequences containing the A194T variant clustered together (Bayes factor, BF, support >100; *n* = 4 sequences). Sequences with this RAM were all from Indonesia, reported by a study exploring HBV genetic diversity.^50^ Some sequences containing both M204V and L180M formed a cluster in genotype B (BF = 54.99, *n* = 4 sequences) and some with M204I formed a cluster in genotype D (BF > 100, *n* = 3 sequences). In genotype C, there were clusters of RAMs S106C (BF > 100, *n* = 5 sequences), R153Q (BF > 100, *n* = 3 sequences) and I129L (BF > 100, *n* = 34 sequences). Clustering of sequences with RAMs might suggest an emerging sublineage of treatment‐resistant virus, although the numbers in each case are small.

### 3.8 Evolution of sequences with RAMs/VEMs

Most sequences with RAMs/VEMs in our analysis were published after the approval of NAs/HBV vaccine, as a result of widespread improvements and availability of sequencing that have arisen in parallel with roll out of drugs and vaccine. However, four sequences (KF214668, KF214671, KF214673 and KF214676) with RAM I269L and one sequence (KF214659) with VEM S/T143M were sampled from Asia in 1963, and one sequence (HQ700441) with RAM L180M was sampled from Oceania in 1984, demonstrating that relevant mutations can arise without exposure to treatment or vaccination.

We performed ML molecular clock analysis for full data sets of sequences of genotypes A–E. However, only genotype C had clusters that had at least two isolates with the same resistant mutations with a single common ancestor as shown in Table 1. The estimated time of emergence of branches with RAMs M204V+L180M was around the year 1945 (95% HPD 1897–1971). Branches with VEM G145R were estimated to emerge around the year 1930 (95% HPD 1866–1958). Importantly, in both cases, the higher bound of the 95% HPD interval of the TMRCA of these clusters, which likely correspond to the lower bound of the estimate of the age of these mutations, precedes the introduction of NAs and the HBV vaccine. The results we obtained from ML molecular clock analysis and BEAST analysis were consistent.

**TABLE 1:** Estimated time of the most common recent ancestor (TMRCAs) (and 95% HPD) of branches with specific RAMs/VEMs on molecular clock trees

Genotype	RAMs/VEMs	Cluster of isolates with specified RAMs/VEMs	Estimated TMRCAs (95% HPD)
C	M204V+L180M	FJ032355	1945 (1897, 1971)
		FJ386620	
	G145R	KU964229	1930 (1866,1958)
		KU964230	

Note

Only genotype C is displayed because it had at least two isolates with the same resistant mutations with a single common ancestor.

Abbreviation: HPD, Highest Posterior Density.

## 4 DISCUSSION

### 4.1 Novel findings and comparison with previous literature

We describe the global prevalence of drug and vaccine resistance in HBV across genotypes and geographical regions and explore the evolution of these mutations using phylogenetic analysis, in order to develop insights into the origins and distribution of drug resistance. Based on this analysis, HBV drug and vaccine resistance are uncommon, with the highest frequency of individual or combined mutations that are well known to cause resistance being ~4%, and the majority being <1%. These mutations are distributed across various continents and genotypes, with the most frequent RAMs/VEMs identified in genotype C, concordant with previous studies from China.^51^, ^52^ We show that these mutations are not only driven by exposure to drug or vaccine but are likely to have been present in some sequences pre‐dating therapy and immunization. More studies representing all genotypes are needed, alongside careful correlation with clinical evidence of drug or vaccine resistance.

M204I/V is one of the best recognized drug resistance motifs in HBV and had the highest overall prevalence of 3.8% within our sequence database. A previous meta‐analysis estimated the prevalence of M204I/V as 4.9% among >12,000 treatment‐naïve individuals,^25^ and another review reported a prevalence of M204I/V of 5.9% among 8435 treatment‐naïve individuals.^9^ These reviews reported prevalence as the proportion of sequences with mutations, without accounting for closely related sequences (thus may include multiple sequences from a single individual). In contrast, we used full‐length HBV sequences and excluded identical sequences, which might explain the lower prevalence we report.

We reported the prevalence of ETV resistance as 2.4%, which is slightly higher than the prevalence of 1.7% reported from a large survey carried out in China among 1223 treatment‐experienced patients, and a prevalence of 1.2% reported from a longitudinal study that followed 108 HBV‐infected treatment‐naïve individuals for five years.^26^, ^53^ Unlike these two studies, we took a lenient approach by reporting the overall prevalence of ETV resistance considering sequences with RAMs M204I/V+L180M, with or without an additional compensatory mutation. These two are always present in ETV resistant variants and are the main ETV RAMs reported in published HBV treatment guidelines.^10^, ^54^

We estimated the overall prevalence of TFV resistance to be between 0.04% and 0.2%. There have been few studies that have reported on TFV resistance^8^ and more robust data are still needed to define HBV resistance in order to guide better estimation of the prevalence of relevant RAMs.

The global prevalence of the VEM G145A/R in our data was 1.3%, which is comparable to a previous report across genotypes A–F.^55^ A study carried out in Italy reported a higher prevalence of 3.1%, in a cohort dominated by genotype D infection.^56^ Regional differences might explain the difference in prevalence. However, the majority of individuals with this mutation from the Italian study were immunocompromised, and the time course of vaccination and infection was not certain.

### 4.2 Relationship between genotype and drug or vaccine resistance

The prevalence of RAMs/VEMs across different regions is influenced by the predominant genotype, but may also relate to different patterns of drug or vaccine exposure in the population. For example, T118A/R/V, A128V and D144A/E/G/N variants are more common in Europe, which may relate to better vaccine coverage^57^ that drives the selection of resistant variants. Some polymorphisms that have been described in association with resistance are wild type in certain genotypes, which might indicate that these genotypes are more susceptible to the development of clinically significant drug resistance. For example, TFV resistance might be selected more easily in genotype A given that RAMs H126Y and R/W153W are wild type in this genotype.^58^

### 4.3 Phylogenetic analysis of selected RAMs and VEMs

We provide evidence that RAMs can arise without exposure to treatment/vaccine, showing that certain RAMs emerged prior to the NAs and vaccine era. Using phylogenetic dating, we estimate that RAMs M204V and L180M, and VEM G145R were already present around the mid 20th century. Although these estimates have wide confidence intervals, their upper bounds precede the time of introduction of NAs and the HBV vaccine. A previous study estimated the origin date of HBV genotype D in Iran as 1894 (95% HPD 1701–1957),^47^ and the root age of genotype A polymerase sequences are estimated as the year 955 (95% HPD 381–1482).^46^ A study that analysed 167 full‐length genotype E sequences, estimated the TMRCA to be 174 years (95% HPD 36–441).^59^ Similar to our analysis, these studies used an uncorrelated relaxed lognormal clock which is reported to the best fitting clock.^46^, ^47^, ^59^ However, given the differences in the substitution models, and with some studies using sequences for just a single gene, direct comparison of the estimated TMRCA generated by these studies and our analysis is challenging.

### 4.4 Selection vs. transmission of drug resistance

Most RAMs were randomly distributed across the branches of HBV phylogenetic trees, which suggests that these polymorphisms are being selected independently in individual hosts (parallel evolution^60^) rather than becoming fixed and disseminated from a founder strain. The high viral replication and mutation rate of HBV can result in amino acid substitutions at sites of resistance, leading to the stochastic emergence of drug RAMs even in individuals who have not been exposed to treatment.^61^, ^62^, ^63^ Individuals can also be infected with HBV strains containing drug RAMs which could significantly comprise virological response to therapy, as has been shown in HIV.^64^

### 4.5 Impact of RAMs/VEMs on liver disease

Given the diverse roles and functions of HBV proteins, and the overlapping nature of the viral genome, mutations occurring within one gene can have varied influences, both within the gene where they are selected and in overlapping regions.^65^ The strongest associations between viral sequence and the evolution of hepatocellular cancer (HCC) are in the X gene and the overlapping basal core promoter region, which are not overlapped by RT or surface gene. However, there are some data to suggest that RAMs and VEMs may influence the development of inflammatory or fibrotic liver disease, and/or HCC. For example, RAMs in HBV reverse transcriptase, A181T/V, M204I and M204V, cause corresponding changes in the overlapping region of HBsAg (W172 stop, W196S/L/Stop and I195M, respectively).^66^, ^67^ S‐gene mutations have been associated with HCC development, potentially because stop codons contribute to an accumulation of surface protein within hepatocytes.^68^

While evidence is lacking for a mechanistic relationship between the presence of common RAMs such as M204I/V+L180M and HCC, an association has been reported.^9^ It is possible that alterations in viral replicative capacity can underpin changes in pathogenicity,^8^, ^9^ but more work is required to determine the extent to which RAMs and VEMs may have functional significance in disease outcomes beyond that of drug or vaccine escape.

### 4.6 Caveats and limitations

The major constraint in this work is the relative lack of HBV sequence data; given the huge global burden of infection, there is a striking lack of high‐quality sequence data available in the public domain, and a minority that represents the full‐length viral genome. As our sequences were obtained from GenBank, metadata on individual characteristics and treatment exposure were not available. Our analyses may not be representative, given the selective nature of generating sequencing data, that is disproportionately representing certain populations and regions that have access to sequencing platforms, and selecting samples for sequencing that contain high viral load.^69^ Drug‐resistant sequences may be over‐represented, given that virus suppressed by drug therapy is not accessible for sequencing and individuals with break‐through viraemia on treatment are more likely to have samples submitted for sequence analysis.

Phylogenetic dating in HBV is challenging. The overlapping reading frames of the viral genome raises controversies around its evolution rates. HBV sequences lack temporal signal thus making it challenging to reliably date HBV evolution using molecular clock methods. In addition, estimation of TMRCA uses sample collection dates obtained from GenBank, which may not be accurate.

While Asia and Africa are known to have the highest prevalence of chronic HBV infection worldwide, estimated at 6.2% and 6.1%, respectively,^70^ 74% (2109/2838) of sequences included in this analysis were from Asia and only 10% (277/2838) from Africa. This low representation of sequences highlights HBV as a neglected disease, with very few individuals diagnosed and linked to care.^71^ In addition, the influence of the widespread use of antiretroviral drugs (frequently containing 3TC and/or TFV) on suppression and/or emergence of drug resistance is not yet understood.

In conclusion, despite the availability of effective prevention and treatment strategies for HBV infection, emergence of RAMs and VEMs may pose a challenge to the achievement of the United Nations sustainable development goals for elimination by 2030. Going forward, enlarged sequencing data sets, collected together with treatment histories and clinical data, will be essential to develop an understanding of the distribution, nature and significance of drug resistance at an individual and population level.

## CONFLICT OF INTEREST

No competing interests were disclosed.

## **AUTHOR** **CONTRIBUTIONS**

JM and PCM conceived the study. JM and MAA assimilated data. JM, TIV and MAA analysed the data. JM, TIV and PCM wrote the manuscript. JM, TIV, EB, MAA, OP and PCM revised the manuscript. All authors have read and approved the manuscript.

## Supporting information

Supplementary Material

## Data Availability

Data not publicly available. Pre‐print submitted to bioRxiv.
