# Supplementary material for: Global prevalence and phylogeny of hepatitis B virus (HBV) drug and vaccine resistance mutations
Source: J Viral Hepat. 2021 May 7;28(8):1110–20. doi: 10.1111/jvh.13525 (PMC8581767; doi:10.1111/jvh.13525)
Supplement: Supplementary file 1 — Supplementary Material [file JVH-28-1110-s001.docx]

**Supplementary Figures**


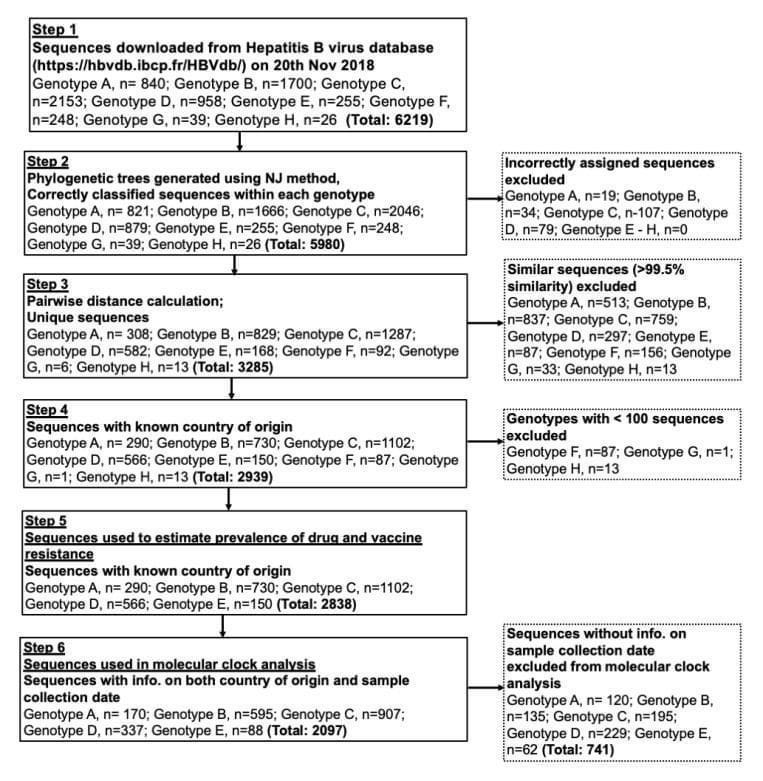
**Suppl Fig 1: Flow diagram showing data curation process of sequences downloaded from a public database (**[**https://hbvdb.ibcp.fr/HBVdb/**](https://hbvdb.ibcp.fr/HBVdb/)**) included in the analysis of the global prevalence and evolution of hepatitis B virus (HBV) drug resistance associated mutation (RAMs) and vaccine escape mutations (VEMs).**

**
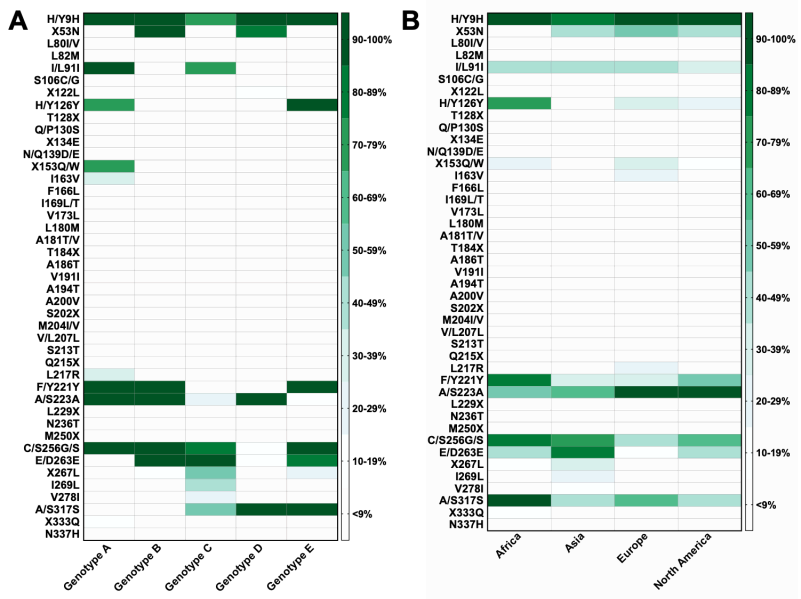
Suppl Fig 2: Global prevalence of hepatitis B virus (HBV) drug resistance associated mutations (RAMs) obtained from analysing 2838** **HBV sequences with information on country of origin, downloaded from a public database (https://hbvdb.ibcp.fr/HBVdb/). A.** Prevalence of polymorphisms across genotypes; **B.** Prevalence of polymorphisms across continents.

X53N represents V/N/S/T53N; X122L represents I/F/H/L/N/Y122L; T128X represents T128A/I/N; X134E represents D/H/N134E; X153Q/W represents Q/R/W153Q/W; T184X represents T184A/C/F/G/I/L/M/S; S202X represents S202C/G/I; Q215X represents Q215E/H/P/S; L229X represents L229G/F/V/W; M250X represents M250I/L/V; X267L represents H/L/M/Q267L.

**
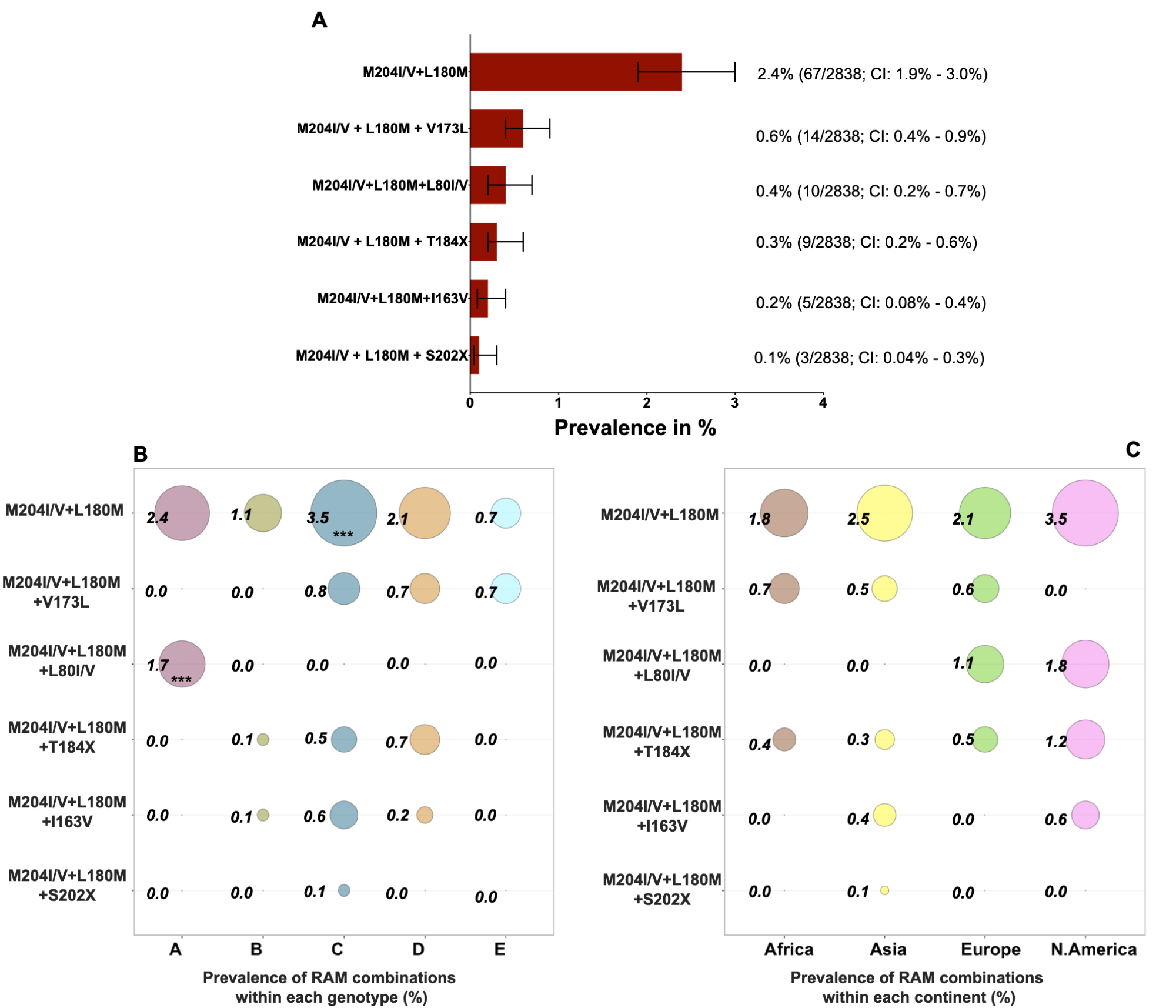
**

**Suppl Fig 3: Global prevalence of hepatitis B virus (HBV) entecavir (ETV) resistance associated mutations (RAMs) obtained from analysing 2838** **HBV sequences with information on country of origin, downloaded from a public database (https://hbvdb.ibcp.fr/HBVdb/). A.** Overall prevalence of ETV RAMs. **B.** A bubble plot showing the prevalence of ETV RAMs within each genotype (genotype A n=290; Genotype B n=730; Genotype C; n=1102; Genotype D n=566 and Genotype E n=150). **C.** A bubble plot showing the prevalence of ETV RAMs within each continent (Africa n=277; Asia n=2109; Europe; n=187 and North America n=170). Numbers next to the circles are prevalence (%) of individual RAMs in each genotype/continent. The asterisks (***/**/*) within certain circles indicate RAMs that have a higher prevalence within the specified genotype/continent compared to the prevalence of that RAM in other genotypes/continents and is statistically significant. *** p value <0.001; **p value < 0.005; *p value <0.05. Bars show 95% confidence intervals. T184X represents T184A/C/F/G/I/L/M/S and S202X represents S202C/G/I/R


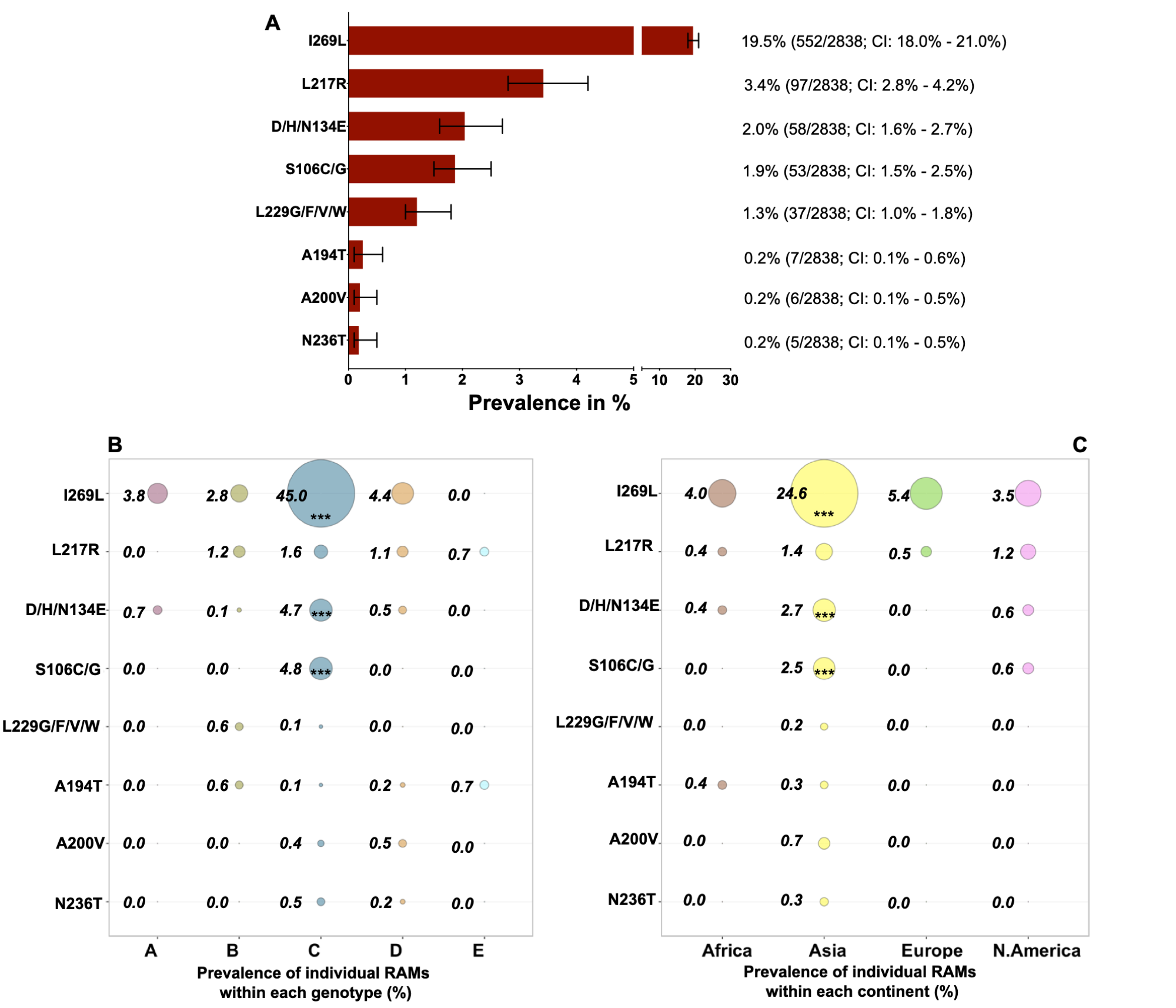


**Suppl Fig 4: Global prevalence of hepatitis B virus (HBV) tenofovir (TFV) resistance associated mutations (RAMs) obtained from analysing 2838** **HBV sequences with information on country of origin, downloaded from a public database (https://hbvdb.ibcp.fr/HBVdb/). A.** Overall prevalence of TFV RAMs. **B.** A bubble plot showing the overall prevalence of TFV RAMs within each genotype (genotype A n=290; Genotype B n=730; Genotype C; n=1102; Genotype D n=566 and Genotype E n=150). **C.** A bubble plot showing the overall prevalence of TFV RAMs within each continent (Africa n=277; Asia n=2109; Europe; n=187 and North America n=170). Numbers next to the circles are prevalence (%) of individual RAMs in each genotype/continent. The asterisks (***/**/*) within certain circles indicate RAMs that have a higher prevalence within the specified genotype/continent compared to the prevalence of that RAM in other genotypes/continents and is statistically significant. *** p value <0.001; **p value < 0.005; *p value <0.05. Bars show 95% confidence intervals.


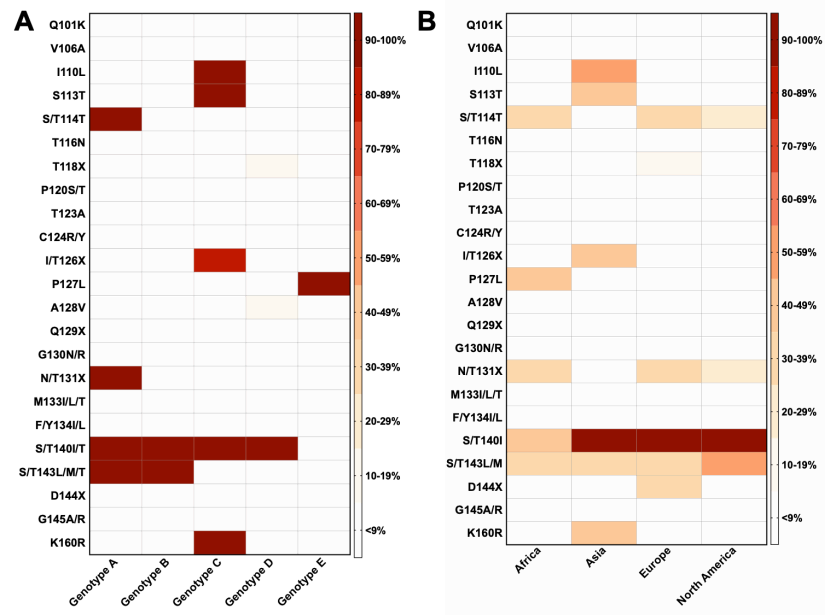


**Suppl Fig 5: Global prevalence of hepatitis B virus (HBV) vaccine escape mutations (VEMs) across genotypes, obtained from analysing 2838** **HBV sequences with information on country of origin, downloaded from a public database (**[**https://hbvdb.ibcp.fr/HBVdb/**](https://hbvdb.ibcp.fr/HBVdb/)**) A.** Showing prevalence of polymorphisms across genotypes; **B.** Showing prevalence of polymorphisms across continents. **Supplementary Tables**

**Suppl Table 1: Hepatitis B virus drug resistance associated mutations (RAMs).** Data obtained from published systematic reviews (1,2,8,9,25,31). Amino acid positions listed in HBV reverse transcriptase protein. 3TC: Lamivudine; ETV: Entecavir; TFV: Tenofovir

| **RAMs** | **3TC** | | | **ETV** | **TFV** | | |
| --- | --- | --- | --- | --- | --- | --- | --- |
|  | **Primary** | **Compensatory** | **Putative** |  | **Clinical and *in vitro* evidence** | **Only clinical evidence** | **Only *in vitro* evidence** |
| H/Y9H |  |  |  |  |  | ✓ |  |
| V/N/S/T53N |  |  | ✓ |  |  |  |  |
| S78T |  |  |  |  | ✓ |  |  |
| L80I/M/V |  | ✓ |  | ✓ |  | ✓ |  |
| L82M |  |  | ✓ |  |  |  |  |
| I/L91I/L |  |  | ✓ |  |  | ✓ |  |
| S106C/G |  |  |  |  | ✓ |  |  |
| T118C/G |  |  |  |  |  | ✓ |  |
| I/F/H/L/N/Y122L |  |  |  |  |  | ✓ |  |
| H/Y126Y |  |  |  |  | ✓ |  |  |
| T128A/I/N |  |  | ✓ |  |  |  |  |
| Q/P130S |  |  |  |  |  | ✓ |  |
| D/H/N134E |  |  |  |  | ✓ |  |  |
| N/Q139D/E |  |  | ✓ |  |  |  |  |
| Q/R/W153Q/R/W |  |  | ✓ |  | ✓ |  |  |
| I163V |  |  |  | ✓ | ✓ |  |  |
| F166L |  |  | ✓ |  |  |  |  |
| I169L/T |  | ✓ |  | ✓ |  |  |  |
| V173L |  | ✓ |  | ✓ | ✓ |  |  |
| P177G |  |  |  |  |  |  | ✓ |
| L180M |  | ✓ |  | ✓ | ✓ |  |  |
| A181T/V | ✓ |  |  | ✓ | ✓ |  |  |
| T184A/C/F/G/I/L/M/S |  | ✓ |  | ✓ |  | ✓ |  |
| A186T |  |  |  | ✓ |  |  |  |
| V191I |  |  | ✓ |  |  | ✓ |  |
| R192P |  |  |  |  |  | ✓ |  |
| A194T |  |  |  |  | ✓ |  |  |
| A200V |  |  | ✓ |  |  | ✓ |  |
| S202C/G/I |  | ✓ |  | ✓ |  |  |  |
| M204I/V/S/Q | ✓ |  |  | ✓ | ✓ |  |  |
| V/L207I/L |  |  | ✓ |  |  | ✓ |  |
| S213T |  |  | ✓ | ✓ |  |  |  |
| Q215E/H/P/S |  |  | ✓ |  |  |  |  |
| L217R |  |  |  |  | ✓ |  |  |
| F/Y221Y |  |  |  |  |  | ✓ |  |
| A/S223A |  |  |  |  |  | ✓ |  |
| L229G/F/V/W |  |  | ✓ |  | ✓ |  |  |
| N236T |  |  |  |  | ✓ |  |  |
| F249A |  |  |  |  |  |  | ✓ |
| M250I/L/V |  |  |  | ✓ |  |  |  |
| C/S256G/S |  |  | ✓ | ✓ |  | ✓ |  |
| E/D263E |  |  |  |  |  | ✓ |  |
| H/L/M/Q267L |  |  |  |  |  | ✓ |  |
| I269L |  |  |  |  | ✓ |  |  |
| V278I |  |  |  |  |  | ✓ |  |
| A/S317S |  |  |  |  |  | ✓ |  |
| K/Q/T333Q |  |  |  |  |  | ✓ |  |
| N337H |  |  |  |  |  | ✓ |  |
|  |  |  |  |  |  |  |  |
| **RAMs common to 3TC & ETV** | | | | I169L/T; S202C/G/I; S213T | | | |
| **RAMs common to 3TC & TFV** | | | | I/L91I/L; Q/R/W153Q/R/W; V191I; A200V; V/L207I/L; L229G/F/V/W | | | |
| **RAMs common to ETV & TFV** | | | | I163V | | | |
| **RAMs common to 3TC & ETV&TFV** | | | | L80I/V/M; V173L; L180M; A181T/V; T184A/C/F/G/I/L/M; M204I/V/S/Q; C/S256G/S | | | |

**Suppl Table 2: Hepatitis B virus vaccine escape mutations (VEMs).** Data obtained from published studies (1,14–16,32–39)**.** Amino acid positions listed in HBV surface protein. VEM: Vaccine escape mutation. HBsAg: Hepatitis B surface antigen.

| **VEMs** | **HBIG** | **VEMs** | **Immune escape** |
| --- | --- | --- | --- |
| Q101K |  |  | ✓ |
| V106A |  |  | ✓ |
| I110L |  |  | ✓ |
| S113T |  |  | ✓ |
| S/T114F/R/T | ✓ |  | ✓ |
| T116A/N |  | ✓ |  |
| T118A/R/V | ✓ |  |  |
| P120A/E/N/Q/S/T | ✓ | ✓ | ✓ |
| R/K122S |  |  |  |
| T123A/N | ✓ |  | ✓ |
| C124R/Y | ✓ |  |  |
| I/T126A/H/I/N/R/S | ✓ | ✓ | ✓ |
| P/T127L/P |  | ✓ | ✓ |
| A128V |  |  | ✓ |
| Q129H/N/R/P | ✓ | ✓ | ✓ |
| G130N/R | ✓ |  | ✓ |
| N/T131I/N/S | ✓ | ✓ | ✓ |
| M133I/L/T | ✓ | ✓ | ✓ |
| F/Y134I/L | ✓ | ✓ | ✓ |
| C137R/Y | ✓ |  |  |
| C138Y |  |  | ✓ |
| C139S | ✓ |  |  |
| S/T140I | ✓ |  | ✓ |
| K141E/I/R | ✓ | ✓ |  |
| P142S | ✓ | ✓ | ✓ |
| S/T143M/L |  | ✓ |  |
| D144A/E/G/H/N | ✓ | ✓ | ✓ |
| G145A/K/R | ✓ | ✓ | ✓ |
| N146S | ✓ |  | ✓ |
| C147S | ✓ |  |  |
| K160R |  |  | ✓ |
|  |  |  |  |
| **VEMs with both phenotypic and experimental evidence** | | K141E/I/R; G145A/K/R | |

**Suppl Table 3: Description of sequences with individual or combination of RAMs that are highly likely to cause resistance to TFV obtained from analysing 2838 HBV sequences with information on country of origin, downloaded from a public database (**[**https://hbvdb.ibcp.fr/HBVdb/**](https://hbvdb.ibcp.fr/HBVdb/)**).** These RAMs combination include ≥ 1 RAMs from the ‘short list’ in combination with ≥ 3 other RAMs from the ‘long list’ as described (8).

| **Sequence ID** | **Individual and combination of mutations that are highly likely to cause resistance to TFV** | **No. of sequences with RAM (%)** | **Continent and number of sequences** | **Genotype**  **and number of sequences** |
| --- | --- | --- | --- | --- |
| GQ358144-7; GQ377536; KT366495; GQ161771 | A194T* | 7 (0.2) | Asia n=6; Africa n=1 | B, n=4;  C, n=1;  D, n=1,  E, n=1, |
| FJ386681 | A181T/V*+N236T | 1 (0.04) | Asia n=1 | B n=1 |
| KJ803809 | S106C*+D134E*+Q267L+I269L*+K333Q | 1 (0.04) | Asia n=1 | C n=1 |
| FJ386579; FJ787470; FJ787471 | S106C*+V173L*+L180M*+M204I/V*+Q267L | 3 (0.1) | Asia n=3 | C n=3 |
| EU939588 | S106C*+L180M*+A200V+M204I*+Q267L | 1 (0.04) | Asia n=1 | C n=1 |
| FJ386623 | S106C*+L180M*+ M204I/V*+Q267L | 1 (0.04) | Asia n=1 | C n=1 |
| FJ386620; JX026877 | S106C*+L180M*+ M204I/V*+I269L | 2 (0.07) | Asia n=2 | C n=2 |
| AB182589 | S106C*+D134E*+Q267L+I269L*+K333Q+N3337H | 1 (0.04) | Asia n=1 | C n=1 |
| JQ040132 | D134E*+L180M*+M204I*+I267L+N337H | 1 (0.04) | Asia n=1 | C n=1 |
| AY641561 | D134E*+I267*L+K333Q+N337H | 1 (0.04) | Asia n=1 | C n=1 |
| EU560439 | D134E*+Q267L+I269L*+K333Q | 1 (0.04) | Asia n=1 | C n=1 |
| JN827423 | R153Q*+V173L*+L180M*+M204V*+I269L*+V278I | 1 (0.04) | Asia n=1 | C n=1 |
| JQ707346 | R153W*+L180M*+M204V*+V207L+L217R* | 1 (0.04) | North America n=1 | A n=1 |
| FJ899789 | R153Q*+Q267L+I269L*+V173L*+N337H | 1 (0.04) | Asia n=1 | C n=1 |
| MF772345 | R153W*+L217R*+V278I+K333Q | 1 (0.04) | Africa n=1 | A n=1 |
| JN257203 | R153Q*+F122L+V278I+K333Q | 1 (0.04) | Africa n=1 | D n=1 |
| KX357637 | V173L*+L180M*+M204V*+V207L | 1 (0.04) | Asia n=1 | D n=1 |
| FJ032355 | V137L+L180M+M204V+Q267L+I269L* | 1 (0.04) | Asia n=1 | C n=1 |
| JN827418; JN827421; MF925409 | V137L*+L180M*+M204V*+I269L*+V278I | 3 (0.1) | Asia n=3 | C n=3 |
| AB697490 | V137L*+L180M*+M204V*+N337H | 1 (0.04) | Asia n=1 | C n=1 |
| FJ787453 | V137L*+ M204V*+ Q267L+I269L* | 1 (0.04) | Asia n=1 | C n=1 |
| FJ386604 | L180M*+A181V*+M204V*+I269L* | 1 (0.04) | Asia n=1 | C n=1 |
| JF828921 | L180M*+T184L+M204V*+L229V*+Q267L+K333Q+N337H | 1 (0.04) | Asia n=1 | C n=1 |
| JF828923; JF828937 | L180M*+T184A/L+M204V*+ Q267L+K333Q+N337H | 2 (0.07) | Asia n=2 | C n=2 |
| EU939564; | L180M*+A200V+M204I*+Q267L | 1 (0.04) | Asia n=1 | C n=1 |
| FJ386653; FJ787455; FJ787456 | L180M*+M204I/V*+L229V*+Q267L | 3 (0.1) | Asia n=3 | C n=2 |
| JN827422; JN827424 | L180M*+M204I/V*+I269L*+V278I | 2 (0.07) | Asia n=2 | C n=2 |
| DQ246215 | L180M*+M204I*+V278I+N337H | 1 (0.04) | Asia n=1 | C n=1 |

* RAMs provided in ‘short list’ described in (8); these RAMs are supported by the highest quality evidence (i.e. isolated from treatment compliant individuals in whom viraemia was not suppressed by TFV and these RAMs were also tested in *in vitro* assays to measure the effect of TFV on viral replication in cell lines)

**Suppl Table 4: Global prevalence of hepatitis B virus (HBV) drug resistance associated mutations (RAMs) that are wildtype amino acid. Prevalence rates were obtained from analysing 2838** **HBV sequences with information on country of origin, downloaded from a public database (https://hbvdb.ibcp.fr/HBVdb/). A.** Identification of RAMs as wildtype in certain genotypes using HBV reference sequences for genotypes A-J. **B.** Prevalence of RAMs that are wildtype amino acid across genotypes. **C.** Prevalence of RAMs that are wildtype amino acid across continents. HBV reference sequences were obtained from a published manuscript (68).

|  |  |  | **HBV RAMs that are wildtype in certain genotypes** | | | | | | | | | | | | |  |
| --- | --- | --- | --- | --- | --- | --- | --- | --- | --- | --- | --- | --- | --- | --- | --- | --- |
| **A** | **Genotype** | **Reference sequence accession number** | **H/Y9H** | **X53N** | **I/L91I** | | **H/Y126Y** | **X153X** | **F/Y221Y** | **A/S223S** | **C/S256S** | **E/D263E** | **X267L** | **A/S317S** | **X333Q** | |
|  | A | FJ692557 | H | - | I | | Y | W | Y | A | S | - | - | - | - | |
|  | B | GU815637 | H | N | - | | - | - | Y | A | S | E | - | - | - | |
|  | C | GQ377617 | H | - | I | | - | - | - | - | S | E | L | S | - | |
|  | D | KC875277 | H | N | - | | - | - | - | A | - | - | - | S | - | |
|  | E | GQ161817 | H | - | - | | Y | - | Y | - | S | E | - | S | - | |
|  | F | HM585194 | H | N | - | | - | - | Y | A | S | - | - | - | Q | |
|  | G | AB056513 | H | - | I | | Y | - | Y | A | S | E | - | - | - | |
|  | H | FJ356715 | H | - | - | | - | - | Y | A | S | E | - | - | - | |
|  | I | AB562463 | H | - | - | | Y | Q | Y | A | S | - | - | - | Q | |
|  | J | AB486012 | - | - | - | | - | - | Y | A | S | - | - | - | - | |
|  |  |  |  |  |  | |  |  |  |  |  |  |  |  |  | |
|  | **Genotypes** | |  |  |  | |  |  |  |  |  |  |  |  |  | |
| **B** | Genotype A | |  |  |  | |  |  |  |  |  |  |  |  |  | |
|  | Genotype B | |  |  |  | |  |  |  |  |  |  |  |  |  | |
|  | Genotype C | |  |  |  | |  |  |  |  |  |  |  |  |  | |
|  | Genotype D | |  |  |  | |  |  |  |  |  |  |  |  |  | |
|  | Genotype E | |  |  |  | |  |  |  |  |  |  |  |  |  | |
|  |  | |  |  |  | |  |  |  |  |  |  |  |  |  | |
|  | **Continents** | |  |  |  | |  |  |  |  |  |  |  |  |  | |
| **C** | Africa | |  |  |  | |  |  |  |  |  |  |  |  |  | |
|  | Asia | |  |  |  | |  |  |  |  |  |  |  |  |  | |
|  | Europe | |  |  |  | |  |  |  |  |  |  |  |  |  | |
|  | North America | |  |  |  | |  |  |  |  |  |  |  |  |  | |
|  |  | |  |  |  | |  |  |  |  |  |  |  |  |  | |
|  | **Key** | |  |  |  | |  |  |  |  |  |  |  |  |  | |
| 81-100% | | |  | | |  |  |  |  |  |  |  |  |  |  |  |
| 61-80% | | |  | | |  |  |  |  |  |  |  |  |  |  |  |
| 41-60% | | |  | | |  |  |  |  |  |  |  |  |  |  |  |
| 21-40% | | |  | | |  |  |  |  |  |  |  |  |  |  |  |
| 0-20% | | |  | | |  |  |  |  |  |  |  |  |  |  |  |

X153Q/W represents Q/R/W153Q/W; X53N represents V/N/S/T53N; X333Q represents K/Q/T333Q.

**Suppl Table 5: Global prevalence of hepatitis B virus (HBV) vaccine escape mutations (VEMs) that are wildtype amino acid. Prevalence rates were obtained from analysing 2838** **HBV sequences with information on country of origin, downloaded from a public database (https://hbvdb.ibcp.fr/HBVdb/). A.** Identification of VEMs as wildtype in certain genotypes using HBV reference sequences for genotypes A-J. **B.** Prevalence of VEMs that are wildtype amino acid across genotypes. **C.** Prevalence of VEMs that are wildtype amino acid across continents.

|  | **HBV RAMs that are wildtype in certain genotypes** | | | | | | | | | | | | | | | | | | | | | | | | |
| --- | --- | --- | --- | --- | --- | --- | --- | --- | --- | --- | --- | --- | --- | --- | --- | --- | --- | --- | --- | --- | --- | --- | --- | --- | --- |
| **A** | **Genotype** | **Reference sequence accession number** | **I110L** | **S113T** | | **S/T114T** | | | | **I/T126I** | | **P/L127L** | | **N/T131N** | | | | | **S/T140T** | | | | | **S/T143T** | **X160R** |
|  | A | FJ692557 | I | - | | T | | | | - | | - | | N | | | | | T | | | | | T | - |
|  | B | GU815637 | I | - | | - | | | | - | | - | | - | | | | | T | | | | | T | - |
|  | C | GQ377617 | L | T | | - | | | | I | | - | | - | | | | | T | | | | | - | R |
|  | D | KC875277 | I | - | | - | | | | - | | - | | - | | | | | T | | | | | - | - |
|  | E | GQ161817 | I | - | | - | | | | - | | L | | - | | | | | - | | | | | - | - |
|  | F | HM585194 | L | - | | T | | | | - | | L | | - | | | | | - | | | | | - | - |
|  | G | AB056513 | I | - | | - | | | | - | | - | | N | | | | | T | | | | | - | - |
|  | H | FJ356715 | L | - | | T | | | | - | | L | | - | | | | | T | | | | | - | - |
|  | I | AB562463 | I | - | | - | | | | - | | - | | N | | | | | T | | | | | - | - |
|  | J | AB486012 | L | - | | T | | | | I | | - | | - | | | | | T | | | | | - | - |
|  |  |  |  |  | |  | | | |  | |  | |  | | | | |  | | | | |  |  |
|  | **Genotypes** | |  |  | |  | | | |  | |  | |  | | | | |  | | | | |  |  |
| **B** | Genotype A | |  |  | |  | | | |  | |  | |  | | | | |  | | | | |  |  |
|  | Genotype B | |  |  | |  | | | |  | |  | |  | | | | |  | | | | |  |  |
|  | Genotype C | |  |  | |  | | | |  | |  | |  | | | | |  | | | | |  |  |
|  | Genotype D | |  |  | |  | | | |  | |  | |  | | | | |  | | | | |  |  |
|  | Genotype E | |  |  | |  | | | |  | |  | |  | | | | |  | | | | |  |  |
|  |  | |  |  | |  | | | |  | |  | |  | | | | |  | | | | |  |  |
|  | **Continents** | |  |  | |  | | | |  | |  | |  | | | | |  | | | | |  |  |
| **C** | Africa | |  |  | |  | | | |  | |  | |  | | | | |  | | | | |  |  |
|  | Asia | |  |  | |  | | | |  | |  | |  | | | | |  | | | | |  |  |
|  | Europe | |  |  | |  | | | |  | |  | |  | | | | |  | | | | |  |  |
|  | North America | |  |  | |  | | | |  | |  | |  | | | | |  | | | | |  |  |
|  |  | |  | |  | | |  | | |  | |  | | |  | |  | | |  | |  |  |  |
|  | **Key** | |  | |  | |  | | |  | | |  | |  | |  | | |  | |  |  |  |  |
| 81-100% | | |  | | | | | |  |  |  |  |  |  |  |  |  |  |  |  |  |  |  |  |  |
| 61-80% | | |  | | | | | |  |  |  |  |  |  |  |  |  |  |  |  |  |  |  |  |  |
| 41-60% | | |  | | | | | |  |  |  |  |  |  |  |  |  |  |  |  |  |  |  |  |  |
| 21-40% | | |  | | | | | |  |  |  |  |  |  |  |  |  |  |  |  |  |  |  |  |  |
| 0-20% | | |  | | | | | |  |  |  |  |  |  |  |  |  |  |  |  |  |  |  |  |  |

X160R represents K/R160R.

**Suppl Methods: Phylogenetic dating using Bayesian Evolutionary Analysis Sampling Trees (BEAST).**

We performed molecular clock phylogenetic analyses to estimate the times of emergence of mutations of interest, focussed on RAMs V173L, L180M and M204I/V as they are well known to cause (individually or synergistically) resistance to 3TC, ETV and TDF (8), and VEMs G145A/R and K141E/I/R as they have been best described to cause HBV vaccine resistance (11–13). In this analysis we included genotypes that had >50 sequences with associated sampling date information: genotype A (n=170), B (n=594), C (n=906), D (n=336) and E (n=88). We manually inspected sequences for misalignments in AliView program (43) and then excluded codon positions associated with resistance (we excluded all sites listed in **Suppl Tables 1 and 2**) to ensure that parallel evolution RAMs/VEMs does not affect the phylogeny (44). We first identified sequences containing these mutations on the molecular clock tree and then only focused on reporting the time to most recent common ancestor (TMRCA) of two or more sequences that clustered together having the same mutation.

We performed Bayesian Markov chain Monte Carlo (MCMC) analyses using BEAST v.1.10 (69). We used a GTR+G nucleotide substitution model, a coalescent Bayesian Skygrid model with 50 points (70) and the uncorrelated lognormal relaxed molecular clock model. These models were selected because they have performed best in other studies estimating HBV evolution (46,47). TempEst allows quantification of temporal signal by estimating regressing the root-to-tip genetic distance of each sequence in the tree and its sampling date (48). Based on application of TempEst, we estimated the correlation between the dates of the tips of the sequences and the divergence from the root to be 7.8 x 10^-2^, 3.9 x 10^-1^ , 4.3 x 10^-2^ , 2.3 x 10^-2^ and 2.1x 10^-1^ for genotypes A, B, C, D and E, respectively, and thus we elected not to estimate the molecular clock rate as there was insufficient signal in our data. We thus fixed the mean substitution rate to 5.0 x 10^-5^ (SD 4.12 x 10^-6^) and a mean standard deviation of 2.0 x 10^-5^ (SD 4.96 x 10^-7^) subs/site/year, for all genotypes in all subsequent BEAST analyses, as this rate has been estimated before and applied in phylodynamic analyses of HBV (24,49).

To avoid convergence issues, we selected smaller subsamples from each of our alignments, depending on their size, to ensure that alignments used in BEAST analyses were <200 sequences. Thus, Genotype B (total n=564) was split into 3 subsamples, genotype C (total n=906) into 6 subsamples and genotype D (total n=336) into 2 subsamples. We used stratified random sampling, ensuring equal representation of sequences with mutations in each subsample. We ran one BEAST analysis for Genotypes A and E since their full alignments contained <200 sequences.

Two MCMC chains of 100 x 10^6^ generations (10% burn-in) were run with sampling every 10,000^th^ generation for genotypes A and E, and for each subsample of genotypes B, C and D separately. The MCMC chains for analyses of the same genotype were combined using LogCombiner v1.10.4 (69). We inspected convergence of the MCMC analyses using Tracer v.1.7.1 (71) to ensure effective sample size (ESS) >200 for all model parameters. We inferred maximum clade credibility trees using TreeAnnotator v.1.10.0 (69) and visualised them using FigTree v.1.4.4 (69).
